# Association Between Chronic Health Conditions and Quality of Life in Rural Teachers

**DOI:** 10.3389/fpsyg.2019.02898

**Published:** 2020-01-09

**Authors:** Pablo A. Lizana, Gustavo Vega-Fernandez, Lydia Lera

**Affiliations:** ^1^Laboratory of Morphological Sciences, Instituto de Biología, Pontificia Universidad Católica de Valparaíso, Valparaíso, Chile; ^2^Public Nutrition Unit, Institute of Nutrition and Food Technology, University of Chile, Santiago, Chile

**Keywords:** mental health, physical health, quality of life, school teachers, obesity, hypertension

## Abstract

**Aim:**

The school teacher profession has been reported to be associated with an increased rate of health problems that can affect the quality of life (QoL) of teachers. However, there is little information about rural teachers.

**Objective:**

To investigate the associations of obesity, abdominal obesity, and hypertension with the perception of QoL in rural teachers.

**Materials and Methods:**

This cross-sectional study included a representative sample of teachers from eight rural schools in the Valparaíso Region of Chile. Obesity was evaluated by the percentages of fat mass (% FM) and abdominal obesity, and hypertension was recorded. The Short Form-36 Health Survey was administered to subjects to evaluate QoL. A logistic regression adjusted for age and gender was used to associate QoL with chronic health conditions.

**Results:**

Eighty-five percent of teachers presented abdominal obesity, 56% presented obesity evaluated by % FM and 33% presented hypertension. Thirty percent of teachers in the ≥45-year-old age group presented all three chronic conditions simultaneously. Teachers presenting abdominal obesity in the ≤44-year-old age group had lower scores in the mental health component (MCS) (*p* < 0.05) of the survey than the teachers in the ≥45-year-old age group. In addition, teachers in the ≤44-year-old age group who presented obesity scored lower in the MCS than those in the same age group without obesity (*p* = 0.004). The occurrence simultaneous of two and three chronic health conditions increased the risk of reduced MCS significantly (*p* = 0.015, OR 17.0, 95% CI: 1.741–165.90 and *p* = 0.003, OR 82.6, 95% CI: 4.58–1490.70, respectively). In addition, ages under 45 years old were associated with low score of MCS (OR 8.8, 95% CI: 1.565–49.698).

**Conclusion:**

A combination of chronic conditions affects the mental health (MH) of rural teachers. Although the association does not imply causation, these findings support the notion that teachers present early MH problems. This suggests that resources must be made available to detect early mental and chronic health conditions of rural teachers.

## Introduction

According to the international labor organization (ILO) and the world health organization (WHO), a good occupational health (OH) and quality of life (QoL) standard is achieved with the promotion and maintenance of mental, physical and social well-being in all workplaces (OIT, 2003; Bagtasos, 2011). In this sense, one of the professions associated with a large decrease in OH worldwide is the school teacher (Travers and Cooper, 1993; Johnson et al., 2005; Innstrand et al., 2011); teaching is associated with significant increases in the deterioration of mental health (MH) and physical discomfort during the practice of the profession (Porto et al., 2006; Bauer et al., 2007; Fernandes and Rocha, 2009; Jurado et al., 2019), as well as subsequent psychosocial deterioration (Bringi and Ranbhare, 2015) and QoL disorders due to occupational stress (Yang et al., 2009). It should be noted that one of the problems that contributes to further deterioration of the QoL is the considerable workload outside of the corresponding schedule (OIT, 2003; Bravo, 2005). The United Nations educational, scientific, and cultural organization (UNESCO) has reported similar conditions in Latin American countries such as Mexico, Chile, Peru, Argentina, Ecuador and Uruguay; Chile is ranked first for teachers who work more than 40 h per week (Cuenca et al., 2005).

Some results in Chile reflect the global situation, where the teaching workload is related to high psychosocial and physical risks and causes a considerable deterioration of the QoL (Cabrera et al., 2003; Cuenca et al., 2005). It is also important to point out that these professionals have a higher disease recurrence rate than the country average (Chávez, 2009), an aspect that has also been reported in other countries (Unterbrink et al., 2008; Bogaert et al., 2014).

The population of teachers in Chile belongs almost entirely to the age ranges of 25–44 and 45–64 years old, which are two of the four age categories studied by the National Health Survey of Chile (Ministerio de Salud de Chile, 2010a). The majority of the working population, particularly those that present diverse health risks due to the prevalence of some chronic non-communicable diseases (CNCDs) related to nutritional status and body composition, is concentrated in these two age ranges. For example, obesity according to body mass index (BMI) (≥25) has a considerable prevalence of 72.26% in both age categories, and a prevalence of 43.8% for high blood pressure has been reported for those 45–64 years old (Ministerio de Salud de Chile, 2010a). In this sense, teachers ≥45 years old have a lower working capacity than teachers ≤45 years old (Freude et al., 2005). Therefore, identifying factors that influence health by age category is relevant for the development of public policies.

In Chile, the rates of overweight, obesity, and abdominal obesity are higher in women from rural areas than in women from urban areas (Ministerio de Salud de Chile, 2010a), which is an important factor since females are mainly employed in the teaching profession (Cabrera et al., 2003; Cuenca et al., 2005). In addition, chronic conditions significantly affect the perception of QoL (Vinaccia and Orozco, 2005; Trevisol et al., 2011; Pimenta et al., 2015), and therefore a decreased QoL may also be present in the population of teaching professionals. However, among the studies on Chile’s workforce, there have been no reports of research on CNCDs in teachers and the impact of these conditions on the perception of QoL in rural teachers.

## Materials and Methods

### Participants

The target population was all teachers working in eight rural primary schools (*N* = 97) in rural establishments in the communities of Calera (32°47’0”S, 71°13′0″W) and Hijuelas (32°48′0″S, 71°10’0”W), Valparaíso, Chile (MINEDUC, 2016). To calculate the sample size, the variable with the greatest variance for this group was selected according to the literature published at the time of planning of this study. The sample was determined with the variable BMI (Ministerio de Salud de Chile, 2010a) for adults of the Valparaíso region. The sample was calculated with 95% confidence and a 5% error. The minimum sample size was calculated as 69 teachers.

Sampling was conducted between April and July 2016. Eighty teachers agreed to participate in the study. The final sample consisted of 70 teachers; 10 teachers were excluded for the following reasons: incomplete data (5), pregnancy (1), and sick leave at the time of the evaluation (4). The teachers presented ages ranging from 24 to 64 years, with an average age of 40.78 ± 12.57 years; 68.6% of the teachers were women. All teachers were evaluated in the same educational establishments during morning sessions.

### Instruments

#### Anthropometry, Body Composition, and Cardiovascular Risk

The weight and height of the subjects were evaluated to determine the BMI (height in meters/kilograms squared), which was categorized as underweight (BMI < 18.5 kg/m^2^), normal weight (18.5 kg/m^2^ ≤ BMI < 25 kg/m^2^), overweight (25 kg/m^2^ ≤ BMI < 30 kg/m^2^), or obese (BMI ≥ 30 kg/m^2^). To evaluate obesity based on the percentage of fat mass (% FM), a bioelectrical impedance device was used (TANITA BC 420 SMA, Tanita, Tokyo, Japan); the subjects were asked not to carry metallic objects, drink alcohol 48 h before the evaluation, perform intense exercise 12 h before the evaluation, eat or drink, especially caffeine or diuretics, 4 h before the evaluation, and were asked to urinate before the evaluation. To classify obesity by % FM, the recommendation by the “American Society of Endocrinologists” was adopted; the cutoff points were >35% in women and >25% in men (Flegal et al., 2012).

The waist circumference (abdominal fat marker) was measured abdominally at the level of the iliac crest and after a normal exhalation while the subject was in a standing position. The tape measure (Lufkin^TM^) was adjusted without compressing the structures. The protocol was outlined in the Clinical Guidelines for Obesity of the United States National Institute of Health (NIH) and was replicated in the National Health Survey from Chile 2003 and 2009–2010 (Ministerio de Salud de Chile, 2010a). The suggested cutoff points were ≥102 cm for men and ≥88 cm for women.

Blood pressure was measured using automated sphygmomanometers (OMRON HEM-705CPINT; Omron Co., Kyoto, Japan). The evaluation protocol involved placing the subject in a sitting and relaxed position for at least 5 min with the left upper limb supported on a table at the level of the heart; the cuff was then placed and adjusted to the perimeter of the subject’s arm. A classification of high blood pressure was a systolic pressure ≥ 140 mmHg and a diastolic pressure ≥ 90 mmHg, according to the clinical guidelines of arterial hypertension outlined by the Ministry of Health of Chile (Ministerio de Salud de Chile, 2010b). For subjects who presented high blood pressure, the procedure was repeated after a ten-minute rest, and the average of three readings performed at an interval of 2 min was considered, similar to the procedure performed in the National Health Survey 2009–2010 (Ministerio de Salud de Chile, 2010a).

#### Quality of Life Questionnaire

The QoL of the teachers was evaluated using the Short-Form 36 Health Survey (SF-36) questionnaire, which is an instrument developed in the United States to evaluate QoL-related health in adults (Ware and Sherbourne, 1992); the SF-36 has been adapted syntactically and semantically for the Chilean population (Olivares, 2006). The questionnaire includes 36 items measured on a Likert scale. The 36 items are grouped into 8 health topics: physical function (FF), physical role (PR), body pain (BP), general health perception (GH), vitality (VT), social function (SF), emotional role (ER), and MH. The 8 topics can be categorized into two components, the physical component summary (PCS) and the mental component summary (MCS). For each scale, T scores, with an average of 50 and a standard deviation of 10, were calculated (McDowell and McDowell, 2006).

### Procedure

Procedures for anthropometric measurements, body composition analysis using bioimpedance, and the SF-36 questionnaire were orally explained to the teachers. In addition, the subjects signed an informed consent form to indicate that they understood the scope of the investigation. Participation in the study was voluntary and the anonymity of teachers was guaranteed. This study was approved by the Ethics Committee of the Pontificia Universidad Católica de Valparaíso in accordance with the Declaration of Helsinki (The World Medical Association, 2009).

#### Data Analysis

Statistical analyses were performed using STATA 15 software (2017, Stata Corp. LLC, College Station, TX, United States). In this cross-sectional study, descriptive analyses are presented as the mean, standard deviation (mean ± SD) and percent according to age category (≤44 years old and ≥45 years old), cutoff scores for age were obtained from the National Health Survey of Chile of Chile (Ministerio de Salud de Chile, 2010a). Comparisons of anthropometric measurements, body compositions and each item of the QoL questionnaire between the groups were carried out using specifics test (*t*-test or its non-parametric equivalent according to Shapiro Wilk’s normality tests). The chi-square (χ2) test was used to evaluate the associations between obesity by BMI, obesity by % FM, abdominal obesity and blood pressure by age category, as well as to evaluate their associations with chronic conditions (obesity by % FM, abdominal obesity and arterial hypertension). The 25th percentiles (25p) of each summary factor (MCS and PCS) were used as a cut-point to dichotomize the data. The teachers were classified in low (below 25p) or fair/good (above 25p) categories. Logistic regression analysis was performed to estimate the association between low scores of MCS and PCS factors with chronic conditions adjusted by age and gender. To verify the accuracy of fit of the model, the Hosmer-Lemeshow test was applied.

## Results

### Characteristics of the Subjects

The total number of subjects participating in this study, their sociodemographic characteristics, and their health characteristics, including body composition, hypertension status, and chronic health conditions, are shown in Table 1. The total sample of the evaluated teachers was 70 individuals (68.57%, women), with an average age of 40.79 ± 12.58 years; the age categories of ≤44 years old and ≥45 years old represented 61.42% and 38.57% of the sample population, respectively, resulting in an average age of 31.62 ± 4.94 years for the first group and 55.37 ± 4.53 years for the second group.

**TABLE 1:** Sociodemographic and health characteristics of rural teachers in Valparaiso, Chile stratified by age group.

	**Total sample**	**≤44**	**≥45**	
	**Mean ± SD**	**Mean ± SD**	**Mean ± SD**	***P***
Age (years)	40.79 ± 12.58	31.62 ± 4.94	55.37 ± 4.53	< 0.01^*a*^
Weight (Kg)	71.30 ± 12.14	69.85 ± 13.15	73.61 ± 10.14	0.209^a^
Height (m)	1.61 ± 0.09	1.62 ± 0.08	1.59 ± 0.08	0.132^a^
BMI (kg/m^2^)	27.18 ± 3.99	26.32 ± 4.00	28.55 ± 3.64	0.022^*a*^
*Normal weight*^e^	20 (28.57)	17 (39.53)	3 (11.11)	0.028^*c*∗^
*Overweight*^e^	32 (45.71)	18 (41.86)	14 (51.85)	
*Obese*^e^	18 (25.71)	8 (18.60)	10 (37.03)	
**Waist circumference (cm)**	94.30 ± 10.30	91.83 ± 10.46	98.24 ± 8.87	0.010^*a*^
*Normal waist*^e^	10 (14.28)	9 (20.43)	1 (3.70)	0.045^*c*∗^
*Central obesity*^e^	60 (85.71)	34 (79.06)	26 (96.29)	
Fat mass (Kg)^d^	23.76 ± 8.37	22.24 ± 9.05	26.17 ± 6.62	0.055^a^
Fat mass (%)^d^	32.99 ± 8.75	31.10 ± 9.05	35.99 ± 7.44	0.022^*a*^
*Normal*^e^	31 (44.28)	26 (60.46)	5 (18.51)	< 0.001^*c*^
*Obese*^e^	39 (55.71)	17 (39.53)	22 (81.48)	
FFM (Kg)^d^	48.14 ± 9.20	47.60 ± 8.47	48.99 ± 10.37	0.542^b^
FFM (%)^d^	66.86 ± 9.04	68.89 ± 9.05	63.63 ± 8.18	0.017^*a*^
Systolic	130.29 ± 18.75	123.90 ± 15.11	140.44 ± 19,72	< 0.001^*a*^
Diastolic	75.44 ± 11.34	7.32 ± 10.23	77.22 ± 12.91	0.302^a^
*Normal pressure*^e^	47 (67.14)	34 (79.07)	13 (48.15)	0.008^*c*^
*Hypertension*^e^	23 (32.86)	9 (20.93)	14 (51.85)	
**Chronic condition combination^f^**				
Normal^e^	15 (21.42)	14 (32.55)	1 (3.70)	
One condition^e^	17 (24.28)	12 (27.90)	5 (18.51)	< 0.001^*c*^
Two conditions^e^	28 (40.00)	15 (34.88)	13 (48.14)	
Three conditions^e^	10 (14.28)	2 (4.56)	8 (29.62)	

*BMI, body mass index; SD, standard deviation; FFM, free fat mass (kg & %). (≤44–≥45), age categories (years). *P* < 0.05. ^*a*^*t*-test. ^*b*^Mann-Whitney-Test. ^*c*^Chi–squared. ^*c*∗^Fisher’s exact test. ^*d*^Body components evaluated by bioimpedance (TANITA BC 420SMA). ^*e*^Data are expressed as frequency (percentage). ^*f*^Chronic conditions: hypertension, central obesity, and obesity.*

### Body Composition and Chronic Conditions

The study sample did not present significant differences between the two age categories in terms of weight (*p* = 0.209) and height (*p* = 0.132). Regarding BMI, both age groups showed significant differences (*p* = 0.022), with the second group having the highest BMI (28.55 ± 3.64 vs. 26.32 ± 4.00 kg/m^2^). In turn, the association between age and BMI was also significant (*p* = 0.028); both age groups had a high prevalence of overweight, at 41.86% (≤44 years old) and 51.85% (≥45 years old), with an evident tendency for BMI to increase with age. Additionally, it was observed that the highest prevalence of obesity was found in the older group, at 18.60% vs. 37.03% in the younger group (Table 1).

Waist circumference was significantly greater in the older age group than in the younger age group (*p* = 0.010), and a majority (85.71%) of the total sample presented abdominal obesity. In addition, waist circumference associated with abdominal obesity was more prevalent in subjects ≥45 years old (79.6% in the younger age range vs. 96.29% in the older age range, *p* = 0.045).

Fat mass (kg) was not significantly different between the age categories (*p* = 0.055), nor was the variable of fat-free mass (FFM, kg) (*p* = 0.542). However, the results of% FM showed a significant difference (*p* = 0.022) between the age two ranges; % FM was higher in the older age group than in the younger age group (Table 1). In addition, the results suggested a significant association (*p* < 0.001) between the age of the subjects and the presence of obesity, with the prevalence of obesity increasing from 39.53% in the younger group to 81.48% in the older group. The % FFM was significantly different between the age groups and was higher in subjects ≤44 years old than in subjects ≥45 years old (68.89 ± 9.05 vs. 63.63 ± 8.18, *p* = 0.017).

Systolic blood pressure was significantly higher (*p* < 0.01) in the older age group. Diastolic pressure was not significantly different between the two age groups (*p* = 0.302); however, these age ranges had a significant association (*p* = 0.008) with hypertension, which tends to occur with age. The older age group had a hypertension prevalence rate of more than 50%.

In the combined analysis of obesity by % FM, abdominal obesity and hypertension in relation to the presence of one, two, or all three chronic conditions in the respective age groups, significant associations were observed with the presence of all three chronic conditions in the older age category (4.56% vs. 29.62%, *p* = 0.010) (Table 1).

### Perception of Quality of Life

Table 2 shows a comparison of the average scores of each of the eight scales and the two summary measures of the SF-36 survey; results are stratified by age and the presence and absence of each chronic condition. For the subjects in the younger age group, there were significant associations between the presence and absence of abdominal obesity and the QoL scores for both the MCS measures (*p* = 0.001) and four of the eight survey scales (PF, RE, BP, and GH; *p* = 0.046, *p* = 0.018, *p* = 0.001, and *p* = 0.042, respectively), while for the older age group, there were no significant differences. In the overall sample of teachers, significant differences were also shown; Abdominal obesity was associated with a deterioration in the QoL in four of the eight scales of the survey (PF, RE, BP, and GH; *p* = 0.014, *p* = 0.027, *p* < 0.001, and *p* = 0.034, respectively) as well as the MCS (*p* = 0.012). Regarding hypertension, there were no significant differences between age groups for conditions associated with QoL scales. For the chronic condition of obesity in the younger age group, significant differences were found between the presence and absence of obesity for one of the eight QoL scales (RE; *p* = 0.026), as well as for the MCS measure (*p* = 0.004). No significant differences were observed for the older age group. Nonetheless, in the total sample, there were significant differences in obesity for three scales of the QoL survey (PF, RE, and MH; *p* = 0.035, *p* = 0.044, and *p* = 0.036, respectively); however, none were observed for the summary measures.

**TABLE 2:** Comparison of the scores on the eight scales of the SF-36 QoL questionnaire in subjects with obesity, abdominal obesity, and hypertension stratified by age group.

	**Obesity**			**Abdominal obesity**			**Hypertension**		
**QoL**	**Absent**	**Current**	***P*^∗^**	**Absent**	**Current**	***P*^∗^**	**Absent**	**Current**	***P*^∗^**
					
	**Mean ± SD**			**Mean ± SD**			**Mean ± SD**		
**≤44^a^**									
PF	53.83 ± 7.45	51.40 ± 8.93	0.339	55.36 ± 6.76	50.50 ± 8.61	0.046	53.19 ± 7.731	51.66 ± 9.61	0.617
RP	50.75 ± 9.59	48.85 ± 12.07	0.569	50.99 ± 9.50	49.06 ± 11.59	0.556	49.03 ± 10.95	53.67 ± 8.33	0.244
RE	52.56 ± 8.29	45.73 ± 11.14	0.026	53.49 ± 8.64	46.40 ± 10.12	0.018	49.20 ± 10.47	52.33 ± 7.89	0.409
BP	48.33 ± 8.08	48.70 ± 10.08	0.894	44.31 ± 6.85	52.45 ± 8.76	0.001	48.85 ± 8.69	47.07 ± 9.67	0.597
SF	50.10 ± 11.36	45.89 ± 9.76	0.216	51.19 ± 7.73	45.81 ± 12.78	0.104	48.55 ± 10.29	47.99 ± 13.41	0.892
MH	53.05 ± 9.32	48.21 ± 10.31	0.117	53.67 ± 8.25	48.72 ± 10.89	0.101	51.37 ± 8.97	50.25 ± 13.45	0.767
VT	49.51 ± 8.89	45.63 ± 10.71	0.203	49.69 ± 10.48	46.34 ± 8.85	0.263	49.03 ± 7.90	44.00 ± 14.67	0.170
GH	49.62 ± 9.33	50.50 ± 10.50	0.774	46.92 ± 6.97	52.87 ± 11.13	0.042	50.50 ± 9.18	47.95 ± 11.86	0.490
**≥45^a^**									
PF	47.44 ± 13.78	44.96 ± 10.83	0.663	48.42 ± 8.22	44.15 ± 12.18	0.375	46.39 ± 9.85	44.52 ± 12.58	0.672
RP	46.76 ± 9.69	50.71 ± 9.22	0.399	52.12 ± 7.73	49.08 ± 9.88	0.446	48.66 ± 9.07	51.20 ± 9.59	0.487
RE	53.27 ± 9.50	49.52 ± 10.44	0.469	52.12 ± 9.99	49.41 ± 10.45	0.539	48.45 ± 9.80	51.85 ± 10.65	0.398
BP	51.27 ± 10.90	52.68 ± 11.73	0.809	46.80 ± 5.94	54.78 ± 12.40	0.096	51.96 ± 10.28	52.84 ± 12.71	0.846
SF	51.57 ± 8.42	52.67 ± 8.16	0.789	49.81 ± 9.22	53.59 ± 7.50	0.274	53.59 ± 5.81	51.43 ± 9.81	0.497
MH	51.40 ± 7.68	47.44 ± 10.55	0.439	51.16 ± 8.56	46.92 ± 10.60	0.326	44.42 ± 8.62	51.67 ± 10.35	0.060
VT	49.91 ± 11.12	53.96 ± 9.56	0.413	53.24 ± 11.53	53.20 ± 9.28	0.992	56.41 ± 11.18	50.24 ± 7.47	0.102
GH	52.28 ± 15.00	49.53 ± 9.78	0.610	49.57 ± 14.45	50.23 ± 9.06	0.887	51.28 ± 10.33	48.88 ± 11.16	0.567
**Total sample**									
PF	52.80 ± 8.79	47.77 ± 10.43	0.035	53.44 ± 7.71	47.56 ± 10.77	0.014	51.31 ± 8.81	47.31 ± 11.82	0.116
RP	50.11 ± 9.56	49.90 ± 10.45	0.932	51.30 ± 8.93	49.07 ± 10.70	0.362	48.93 ± 10.37	52.17 ± 9.00	0.205
RE	52.68 ± 8.33	47.87 ± 10.77	0.044	53.11 ± 8.86	47.80 ± 10.26	0.027	49.00 ± 10.19	52.04 ± 9.47	0.234
BP	48.80 ± 8.45	50.94 ± 11.08	0.377	44.99 ± 6.61	53.53 ± 10.52	<0.001	49.71 ± 9.15	50.58 ± 11.73	0.733
SF	50.34 ± 10.83	49.72 ± 9.41	0.797	50.81 ± 8.02	49.41 ± 11.24	0.568	49.95 ± 9.48	50.09 ± 11.19	0.956
MH	52.79 ± 8.98	47.78 ± 10.31	0.036	52.98 ± 8.26	47.88 ± 10.66	0.034	49.45 ± 9.33	51.11 ± 11.38	0.516
VT	49.58 ± 9.07	50.33 ± 10.79	0.757	50.67 ± 10.69	49.52 ± 9.58	0.638	51.07 ± 9.41	47.80 ± 11.00	0.201
GH	50.05 ± 10.18	49.95 ± 9.98	0.968	47.65 ± 9.40	51.65 ± 10.18	0.099	50.72 ± 9.40	48.52 ± 11.18	0.391

*QoL, quality of life; PF, physical function; RP, role limitations due to physical problems; RE, role limitations due to emotional problems; BP, bodily pain; SF, social functioning; MH, mental health; VT, vitality; GH, general health perception; *P* < 0.05. ^∗^*t*- test. ^*a*^≤44–≥45, age categories (years).*

Table 3 shows that% FM and abdominal obesity have significant associations with a poor perception of QoL in the MCS in the younger age group (*p* < 0.005). In addition, abdominal obesity also presented a significant association with the PCS in the ≤44-year-old age group (*p* = 0.037).

**TABLE 3:** Comparison of physical component and mental component summary measures for subjects with three chronic health conditions stratified according to age group.

	**≤44^a^**		**≥45^a^**		**Total sample**	
	**PCS**	**MCS**	**PCS**	**MCS**	**PCS**	**MCS**
			
	**Mean ± SD**	**Mean ± SD**	**Mean ± SD**	**Mean ± SD**	**Mean ± SD**	**Mean ± SD**
**Fat mass (%)**						
Non-obese	47.62 ± 5.75	49.59 ± 8.05	45.34 ± 7.87	51.21 ± 7.78	47.25 ± 6.04	49.85 ± 7.90
Obese	48.56 ± 6.33	42.96 ± 5.23	47.13 ± 5.32	48.90 ± 8.02	47.76 ± 5.75	46.31 ± 7.48
*P*^∗^	0.616	0.004	0.538	0.564	0.723	0.059
**Blood pressure**						
Non-hypertensive	48.14 ± 4.99	47.08 ± 6.66	48.17 ± 4.99	47.76 ± 7.74	48.15 ± 4.93	47.26 ± 6.98
Hypertensive	47.33 ± 9.02	46.55 ± 11.43	45.53 ± 6.26	50.79 ± 8.01	46.27 ± 7.32	49.13 ± 9.48
*P*^∗^	0.752	0.859	0.240	0.327	0.209	0.351
**Abdominal obesity**						
Normal	46.08 ± 5.72	50.68 ± 7.51	45.92 ± 6.13	50.50 ± 7.23	46.04 ± 5.72	50.63 ± 7.30
Obese	49.82 ± 5.66	43.43 ± 6.24	47.17 ± 5.69	48.84 ± 8.28	48.59 ± 5.76	45.93 ± 7.66
*P*^∗^	0.037	0.001	0.615	0.626	0.071	0.012

*PCS, physical component summary; MCS, mental component summary; SD, standard deviation. ^*a*^≤44–≥45, age categories (years). ^∗^Mann-Whitney-Test. *P* < 0.05.*

The results for the association of 25th percentiles of MCS and PCS with chronic health conditions variables adjusted by age and gender are given in Table 4. The association between chronic health conditions and PCS was not significant. However, the occurrence simultaneous of two and three chronic health conditions increased the risk of reduced MCS significantly (*p* = 0.015, OR 17.0, 95% CI: 1.741–165.90 and *p* = 0.003, OR 82.6, 95% CI: 4.58–1490.70, respectively). In addition, ages under 45 years old were associated with low MCS (*p* = 0.014, OR 8.8, 95% CI: 1.565–49.698), but the association with gender was insignificant.

**TABLE 4:** Logistic regressions for the association of 25th percentiles of MCS and PCS with chronic health conditions adjusted by age and gender.

	**MCS**		**PCS**	
	**OR [95% CI]^a^**	***P***	**OR [95% CI]^a^**	***P***
One condition	1.133 [0.063–20.202]	0.932	3.219 [0.508–20.390]	0.215
Two conditions	16.995 [1.741–165.900]	0.015	2.402 [0.383–15.070]	0.940
Three conditions	82.616 [4.579–1490.49]	0.003	1.283 [0.114–4.331]	0.840
Age (≤44 years old)	8.820 [1.565–49.698]	0.014	0.577 [0.165–2.015]	0.389
Gender (female)	0.842 [0.188–3.772]	0.823	0.477 [0.145–1.565]	0.223
Hosmer-Lemeshow	0.606		0.427	

*PCS, physical component summary; MCS, mental component summary. ^*a*^Odds Ratios [Confidence interval]. *P* < 0.05.*

## Discussion

This study found that teachers from rural locations had a high prevalence of CNCDs and that each of the studied CNCDs increased in subjects ≥45 years old. The prevalence of overweight at ages 25–44 and 45–64 years, as reported in the National Health Survey of Chile (Ministerio de Salud de Chile, 2010a), decreased slightly from 44% to 41%. However, we observed an increase in overweight due to BMI (51.85% of the subjects) in the older age group. Regarding obesity measured using BMI, this research showing that the older age group had the highest prevalence (present study 37.03%, 35.8%) were similar to the National Health Survey of Chile (Ministerio de Salud de Chile, 2010a). A high prevalence of overweight and obesity evaluated by BMI in teachers was previously been reported for Chilean urban teachers (Cabrera et al., 2003; Kain et al., 2010), but the results were not stratified by age category. The usefulness of obesity evaluated by BMI is limited because it does not consider differences in corporal components (Prado et al., 2015). For this reason, in the present study, it was decided to define obesity according to % FM. When obesity was measured by % FM, the obesity prevalence in rural teachers in the older age group 81.48%, whereas when obesity was evaluated by BMI, the prevalence in the older age group was 37.03%; therefore, there could be an underestimation of obesity in working-age population studies that use BMI.

Teachers belonging to the second age group (≥45 years old) showed a higher prevalence (51.85%) of high blood pressure than teachers in the same age group at the country level (43.8%) in the National Health Survey of Chile (Ministerio de Salud de Chile, 2010a). This coincides with Cabrera et al. (2003), who also reported a higher prevalence of hypertension in teachers than that in the reference group. Regarding abdominal obesity and its categories stratified by age group in the National Health Survey of Chile (Ministerio de Salud de Chile, 2010a), a prevalence of 62.4% was observed for the group aged 25–44 years and 80.6% for the group aged 45–64 years. These values were far less than the values reported in the present study, with a prevalence of 79.6% in the younger group and 96.29% in the older group. These results indicate that cardiometabolic risk and metabolic syndrome are important factors (Despres et al., 2008).

Chronic conditions in rural teachers were mainly associated with the older age group, but the QoL MCS was affected by age. In Chile, teachers have a high workload that includes lessons and administration, as well as a series of working hours outside the classroom, which may include test review, planning, and educational material preparation. Additionally, tasks such as interacting with parents, solving school problems (inside and outside the classroom), and building relationships with peers, among others, may require an important social or emotional component (Cuenca et al., 2005). Therefore, this work overload may affect QoL, especially the MCS, of teachers, as observed in this study. Bauer et al. (2007) applied a general health questionnaire and found that the MCS was affected in 30% of German urban school teachers. Therefore, it is important to generate mitigation strategies, such as social networks inside and outside the classroom, to promote MH in teachers.

In addition, in this study, a low score in the MCS of the QoL survey was associated with the simultaneous presence of two and three chronic health conditions in rural teachers, even after adjustment for gender. We adjusted for gender because the teachers who participated in this study were mostly women (68.57%), an aspect that coincides with previous studies in Chilean school teachers (Cabrera et al., 2003; Cuenca et al., 2005). The significant associations between the MCS and the chronic conditions indicate that teachers should have ways to prevent and identify MH issues and strategies to improve their health, thus improving their QoL. Interestingly, teachers perceive MCS deterioration but not PCS deterioration, which can be related to the varied high-stress tasks that the teaching profession entails in Chile. Accordingly, Chile has one of the highest medical leave rates in South America (Cuenca et al., 2005), and these medical leave rates are often associated with emotional fatigue, which corresponds to the results of our work, as lower MCS scores were associated with chronic conditions.

The present study has several limitations. The first is typical of cross-sectional research; one of the instruments used, the QoL survey, was based on self-reports, so no causality can be derived from the reported associations. Nevertheless, we obtained a high response rate from teachers. Additionally, although we investigated a representative sample of rural teachers, we worked in only two rural areas in a region of Chile, which may limit the generalization of the study results to other regions. Further studies should include teachers in urban areas who may have other conditions that affect their QoL. Although there are important studies on the QoL of urban teachers (Fernandes and Rocha, 2009; Yang et al., 2009; Pizolato et al., 2013; Nusseck et al., 2018), there are few studies focusing on the health of rural teachers; therefore, the present study has the strength of being a pioneer study involving rural teachers, health and QoL.

Currently, occupational health in Chile confers safety and prevention to workers in general but not specifically to school teachers. Therefore, this research has an important implication in enhance the background on health in rural school teachers, providing a clear picture to establish possible improvements and implementation of public policies in the prevention of mental and physical health of school teachers. In this sense, Arvidsson et al. (2016) suggests a series of measures for teachers at the social, organizational and individual levels. At the organizational and individual level that improve leadership and strengthen school teacher self-efficacy. They also suggest improving coordination between the various stakeholders, the rationalization of administrative tasks and better ergonomic conditions during work, aspects that point to the mental and physical health components. In addition, and given the lack of time that has been reported in Chilean school teachers (Cuenca et al., 2005) should include policies for balancing work life and leisure time, in order to generate healthy living spaces and reduce the prevalence of CNCDs in school teachers.

## Conclusion

In conclusion, chronic health conditions mainly affect the QoL MCS rather than the PCS in rural teachers. This suggests that sufficient resources must be available to detect and manage early chronic conditions in teachers in rural areas since chronic conditions are associated with the MCS. Teaching is an occupation that requires significant mental work, and chronic conditions affecting the MCS could affect work performance and thus affect the students.

## Data Availability Statement

The datasets generated for this study are available on request to the corresponding author.

## Ethics Statement

The studies involving human participants were reviewed and approved by the Pontificia Universidad Católica de Valparaíso. The patients/participants provided their written informed consent to participate in this study.

## Author Contributions

PL designed the study. PL and GV-F performed the measurements, processed the data, drafted the manuscript, and designed the tables. PL and LL performed the statistical analysis. All authors discussed the results and commented on the manuscript.

## Conflict of Interest

The authors declare that the research was conducted in the absence of any commercial or financial relationships that could be construed as a potential conflict of interest.
